# Chimeric antigen receptor T-cell therapy after COVID-19 in refractory high-grade B-cell lymphoma

**DOI:** 10.1007/s12185-024-03711-5

**Published:** 2024-02-13

**Authors:** Kenta Hayashino, Keisuke Seike, Kanako Fujiwara, Kaho Kondo, Chisato Matsubara, Toshiki Terao, Wataru Kitamura, Chihiro Kamoi, Hideaki Fujiwara, Noboru Asada, Hisakazu Nishimori, Daisuke Ennishi, Keiko Fujii, Nobuharu Fujii, Ken-ichi Matsuoka, Yoshinobu Maeda

**Affiliations:** 1 Department of Hematology and Oncology, Okayama University Hospital, 2-5-1 Shikata, Okayama-shi, Okayama, Japan; 2 Division of Blood Transfusion, Okayama University Hospital, 2-5-1 Shikata, Okayama-shi, Okayama, Japan; 3 Center for Comprehensive Genomic Medicine, Okayama University Hospital, 2-5-1 Shikata, Okayama-shi, Okayama, Japan; 4 Division of Clinical Laboratory, Okayama University Hospital, 2-5-1 Shikata, Okayama-shi, Okayama, Japan

**Keywords:** COVID-19, Lymphoma, CAR-T-cell therapy, Molnupiravir

## Abstract

Although chimeric antigen receptor T-cell (CAR-T) therapies have dramatically improved the outcomes of relapsed/refractory B-cell malignancies, recipients suffer from severe humoral immunodeficiencies. Furthermore, patients with coronavirus disease 2019 (COVID-19) have a poor prognosis, as noted in several case reports of recipients who had COVID-19 before the infusion. We report the case of a 70-year-old woman who developed COVID-19 immediately before CAR-T therapy for high-grade B-cell lymphoma. She received Tixagevimab−Cilgavimab chemotherapy and radiation therapy but never achieved remission. She was transferred to our hospital for CAR-T therapy, but developed COVID-19. Her symptoms were mild and she was treated with long-term molnupiravir. On day 28 post-infection, lymphodepleting chemotherapy was restarted after a negative polymerase chain reaction (PCR) test was confirmed. The patient did not experience recurrence of COVID-19 symptoms or severe cytokine release syndrome. Based on the analysis and comparison of the previous reports with this case, we believe that CAR-T therapy should be postponed until a negative PCR test is confirmed. In addition, Tixagevimab−Cilgavimab and long term direct-acting antiviral agent treatment can be effective prophylaxis for severe COVID-19 and shortening the duration of infection.

## Introduction

Chimeric antigen receptor (CAR) T-cell therapy is an innovative immunotherapy for relapsed/refractory B-cell malignancies. CAR-T cells destroy malignant as well as normal B-cells. Therefore, most CAR-T cell recipients suffer from severe and persistent humoral immunodeficiencies and are recommended routine immunoglobulin supplementation [1, 2].

Although the prognosis of coronavirus disease 2019 (COVID-19) has improved significantly with the development of vaccines and treatments, including neutralizing monoclonal antibodies and direct-acting antiviral agents, its prognosis in cancer patients remains poor [3, 4]; particularly, in immunocompromised patients with hematological malignancies. In patients with non-Hodgkin’s lymphoma and CAR-T cell recipients, the overall mortality rate is 30–40% [2, 5, 6]. However, there have been few cases of CAR-T cell recipients infected with severe acute respiratory syndrome coronavirus 2 (SARS-CoV-2) immediately prior to infusion.

Herein, we report the case of a 70-year-old woman with refractory high-grade B-cell lymphoma. Although she was infected with SARS-CoV-2 before lymphodepleting chemotherapy and infusion of lisocabtagene maraleucel (liso-cel), CAR-T therapy was performed without severe cytokine release syndrome (CRS) and aggravation of COVID-19 symptoms.

## Case report

A 70-year-old woman with a history of asthma and three COVID-19 vaccinations presented at a nearby hospital with right cervical lymphadenopathy. Lymph node biopsy revealed a large B-cell lymphoma (LBCL) localized to the cervical lymph nodes (Ann Arbor: Stage II, International Prognostic Index score: 1 point, low risk). Large cells were positive for CD10, CD20, CD79a, BCL-2, and BCL-6, and negative for CD5 and MUM-1. However, she received two cycles of rituximab, cyclophosphamide, doxorubicin, vincristine, and prednisone, but the lymphoma progressed. Repeat biopsy revealed LBCL, and extensive interphase fluorescence in situ hybridization showed the presence of c-MYC and BCL-2 rearrangement. The patient was diagnosed with high-grade B-cell lymphoma with MYC and BCL2 rearrangements.

Although she was treated with one cycle of polatuzumab vedotin, bendamustine, and rituximab, the cervical lymphoma lesion progressed, and new lesions appeared in the mesenteric lymph nodes. As salvage therapy, the patient received four cycles of rituximab, dexamethasone, etoposide, ifosfamide, carboplatin (R-DeVIC), and radiation therapy (40 Gy) for the cervical lesions. At the end of the therapy, computed tomography (CT) showed a decrease in the size of lymphoma lesions, and leukapheresis for CAR-T therapy was performed for primary refractory high-grade B-cell lymphoma. Because the Tixagevimab−Cilgavimab is permitted to administrate as preventive medicine for patients with hematological malignancies receiving treatments in Japan, Tixagevimab−Cilgavimab was administered as prophylaxis for COVID-19 infection two months before the patient was transferred to our hospital.

On day 7 of the 5th cycle of R-DeVIC, the patient was transferred to our hospital for CAR-T therapy. She had an Eastern Cooperative Oncology Group performance status score of 1. Laboratory examinations were as follows: white blood cell count, 5970/μL; neutrophil count, 5731/μL; lymphocyte count, 178/μL (CD4 positive T-cell count, 67/μL; CD8 positive T-cell count, 98/μL; B-cell count, 0/μL; natural killer cell count, 13/μL); hemoglobin level, 8.6 g/dL; platelet count, 279,000/μL; lactate dehydrogenase level, 240 U/L; soluble interleukin-2 receptor level, 461 U/mL; IgG level, 710 mg/dL; IgA level, 176 mg/dL; IgM level, 7.1 mg/dL. CT showed a decrease in the size of cervical lesions and progression of mesenteric lesions when compared with that of the previous month’s findings. Bone marrow aspiration and biopsy, magnetic resonance imaging of the brain, and cerebrospinal fluid analysis showed no evidence of lymphoma. At admission, the patient screened negative for COVID-19 using a polymerase chain reaction (PCR) test.

**Fig. 1:**
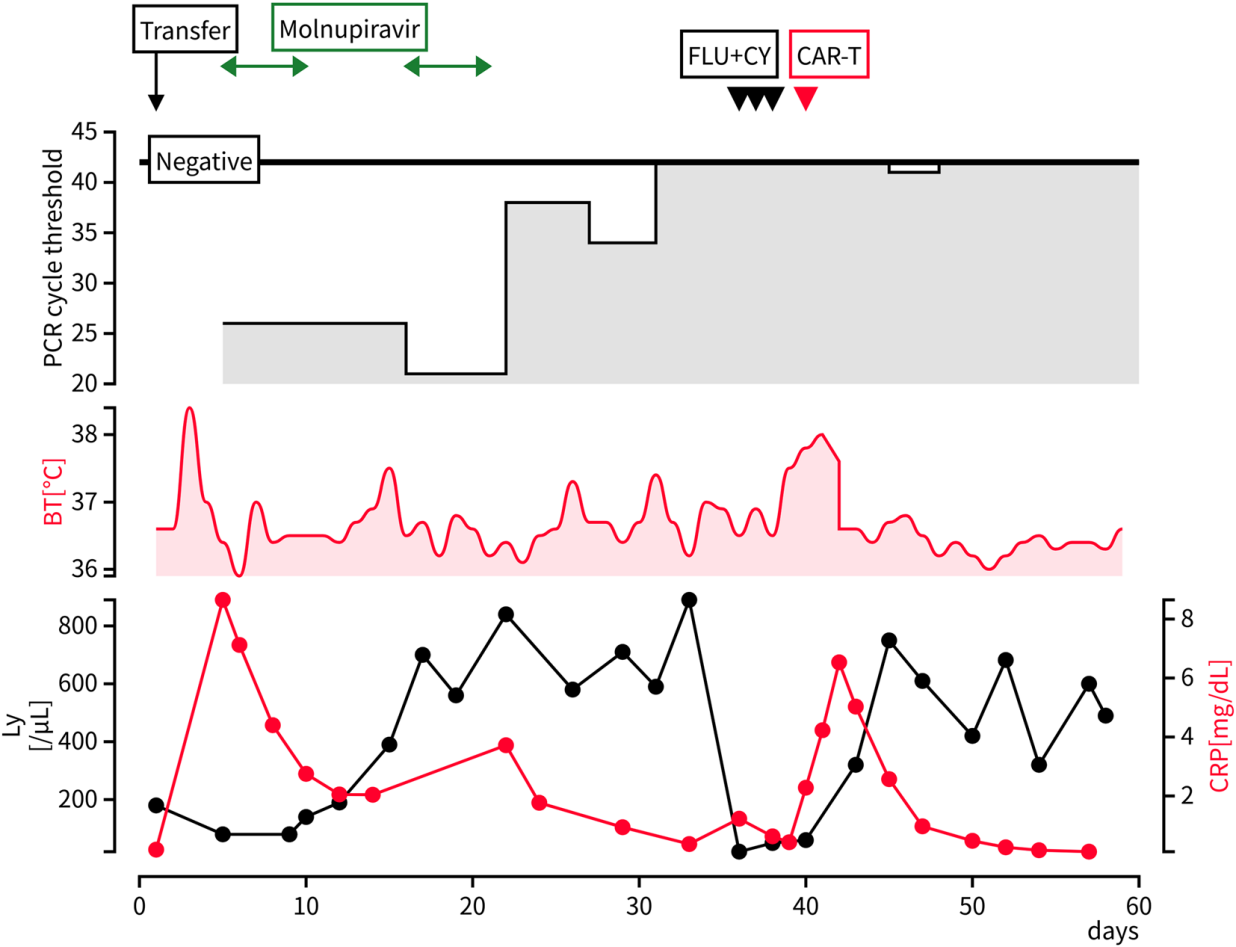
Clinical course of this case. Ly: lymphocyte count, CRP: C-reactive protein (reference range < 0.14 mg/dL), BT: body temperature, FLU: fludarabine, CY: cyclophosphamide, CAR-T: chimeric antigen receptor T

Exactly 3 days after admission, she developed a fever without respiratory symptoms. Laboratory examination revealed a neutrophil count of 40/μL, following which she was treated with cefepime for febrile neutropenia. The fever subsided the following day. On the 5th day of admission, she was screened again using PCR as the medical staff attending to her were infected with SARS-CoV-2. The patient tested positive on PCR; the cycle threshold value was 26. Chest radiography revealed no abnormalities. The patient was immediately transferred to the COVID-19 unit and treated with molnupiravir for 5 days. Although she had no new COVID-19 symptoms, the PCR test was positive (cycle threshold value, 21) 10 days after its onset. CT showed no abnormalities in the lungs. Suspecting COVID-19 symptoms to worsen if CAR-T therapy was initiated during the replication of SARS-CoV-2, the CAR-T treatment plan was suspended. The patient was treated again with molnupiravir for 5 days with her consent. After treating with molnupiravir for the second time, the PCR results remained positive; however, the cycle threshold value improved from 21 to 38. The SARS-CoV-2 anti–S-protein IgG titer (Roche Elecsys Anti-SARS-CoV-2) was elevated to 4675 U/mL. The PCR test was negative on the 26th day after the COVID-19 diagnosis. Cervical and mesenteric lesions were stable during the SARS-CoV-2 infection. On day 28 after infection, lymphodepleting chemotherapy (fludarabine 30 mg/m^2^ and cyclophosphamide 300 mg/m^2^ for 3 days) was administered. CAR-T cells (liso-cel) were infused on day 33. The following day, grade 1 CRS developed, and the patient was treated with acetaminophen. The PCR test was repeated twice a week after infusion, and only one PCR test was positive (cycle threshold value, 41) on day 6 of infusion. CT showed a decreased lesion size on day 20 after CAR-T cell infusion. Although she showed no signs of COVID-19 symptoms or neurotoxicity during hospitalization, she was transferred to a nearby hospital on day 22 because of severe pancytopenia caused by CAR-T therapy (Fig. 1).

## Discussion

Here, we report a case of high-grade B-cell lymphoma in a patient with COVID-19 before CAR-T therapy. Although the patient was infected immediately before the infusion, she did not experience severe CRS, neurotoxicity, or respiratory symptoms. In this case, our findings suggest three important points. First, once a negative PCR test is confirmed, COVID-19 infection may not affect the prognosis irrespective of its occurrence before or after infusion. Secondl Tixagevimab−Cilgavimab could be an effective preventive medicine for patients with lymphoma who plan to be treated with CAR-T. Third, long-term administration of direct-acting antiviral agents might be effective for patients who are immunodeficiency and remain PCR test positive for long time.

Patients with hematological malignancies, including lymphoma, are not expected to benefit from the vaccine compared with healthy individuals because of their severe immunodeficiency, and SARS-CoV-2 infection frequently becomes severe [6–9]. Particularly, CAR-T cell recipients with suppressed humoral immunity frequently experience a severe clinical course [2, 5]. In contrast, few lymphoma cases have been reported in CAR-T cell recipients infected with SARS-CoV-2 immediately before infusion [10–12] (Table 1). In three of the four cases reported, including this case, negative PCR tests were confirmed before infusion, and none of the patients experienced severe CRS or recurrence of COVID-19 symptoms. However, one of the patients was treated with lymphodepleting chemotherapy after a positive PCR test and had to be admitted to the intensive care unit because of acute respiratory distress syndrome before infusion (Table 1: case 2).

**Table 1:** Previous and current cases of chimeric antigen receptor T-cell therapy following COVID-19

Case	Reference	Age/sex	Disease	Vaccine	Tixagevimab-cilgavimab	Lymphocyte count at infection	SARS-Cov-2 IgG before infusion	Days from infection to CAR-T infusion	PCR test at the time of infusion	CAR-T	Outcome of CAR-T	Outcome of COVID-19
1	[10]	55/M	DLBCL	N/A	N/A	N/A	N/A	115	Negative	axi-cel	Complete remission with CRS (grade 2)	No change
2	[11]	60/M	DLBCL transform from FL	No	N/A	0/μL	N/A	34	Positive	axi-cel	Complete remission without any grade of CRS	Transferred to ICU because of ARDS
3	[12]	55/M	Multiple Myeloma	N/A	N/A	About 500/μL	2880U/mL (Mount Sinai)	44	Negative	bb21217(clinical study)	Partial response with CRS (Grade 2)	No change
4	Present case	70/F	High-grade B-cell lymphoma	Three times	Yes	178/μL	4865U/mL (Roche)	33	Negative	liso-cel	Partial response with CRS (grade 1)	No change

*N/A*: not available, *SARS-CoV-2*: severe acute respiratory syndrome coronavirus 2, *COVID-19*: coronavirus disease 2019, PCR: polymerase chain reaction, *M*: male, *F*: female, *DLBCL*: diffuse large B-cell lymphoma, *FL*: follicular lymphoma, *CAR*: chimeric antigen receptor, *axi-cel*: axicabtagene ciloleucel, liso-cel: lisocabtagene maraleucel, *CRS*: cytokine release syndrome, *ICU*: intensive care unit, *ARDS*: acute respiratory distress syndrome

In the opinion of some professionals, if CAR-T cell recipients are infected with SARS-CoV-2 before infusion, CAR-T therapy should be postponed until at least 14 days after symptom resolution and a negative PCR test is confirmed [13]. In this case, CAR-T therapy was postponed until the PCR test was negative. In healthy individuals, the median duration of SARS-CoV-2 RNA shedding is 17 days from symptom onset [14]. Meanwhile, immunocompromised patients shed SARS-CoV-2 for over 2 months [15]. Fortunately, in this case, the PCR test was negative after 1 month, and the lymphoma lesion did not progress during that time. Tixagevimab−Cilgavimab, long-term therapy with molnupiravir, and recovery from lymphocytopenia may have contributed to the rapid recovery from COVID-19.

However, there are other options for reducing the risk and severity of COVID-19. The patient in this case had received three COVID-19 vaccinations before developing high-grade B-cell lymphoma, and about one year had passed from the vaccination. However, patients who receive the vaccination before B-cell depletion scarcely acquire sufficient levels of SARS-CoV-2 neutralizing antibodies [16]. Tixagevimab−Cilgavimab is a SARS-CoV-2 neutralizing monoclonal antibody derived from B-cells isolated from patients with COVID-19. Tixagevimab−Cilgavimab has a half-life of approximately 90 days, which is significantly longer than that of other neutralizing monoclonal antibodies. Although Tixagevimab−Cilgavimab itself cannot prevent infection, it is expected to keep severe SARS-CoV-2 infection at bay for 6 months in patients with B-cell malignancy [7]. Consistent with this report, Tixagevimab−Cilgavimab did not prevent her SARS-CoV-2 infection, but her symptom was very mild. In addition, the immunocompromised host tend to be prolonged viral clearance, and the detection of SARS-CoV-2 persists for 2 months in lymphoma patients [15]. When compared with this report, in this case, virus clearance was relatively early. Based on these two reasons, we thought that Tixagevimab−Cilgavimab was an effective preventive medicine.

Molnupiravir is a small-molecule ribonucleoside prodrug that prevents the replication of SARS-CoV-2. This case was observed during the pandemic era during which time approximately 70,000 people were infected daily with SARS-CoV-2 in Japan. At that time, the Omicron BA.5 virus accounted for most of cases. Molnupiravir effectively reduces Omicron BA.5 virus replication in patients with mild-to-moderate COVID-19. As molnupiravir is effective in both healthy individuals and immunocompromised patients, the patient was treated with molnupiravir [17, 18]. However, immunocompromised patients will likely be severely affected and shed viable SARS-CoV-2 longer than healthy individuals. There are no established therapeutic protocols for immunocompromised patients in terms of drug selection or duration of therapy. There were some reports of patients with hematological malignancies who were refractory to conventional therapy and continued to shed viral RNA. Two of these cases were treated with remdesivir, which inhibits RNA polymerase by a similar mechanism to molnupiravir, repeatedly and for a long time [19, 20]; the other was treated with a combination of nirmatrelvir/ritonavir and molnupiravir for a prolonged period [21]. These atypical treatments resulted in the improvement of persistent COVID-19 infection. Herein, the patient was retreated with molnupiravir as it did not induce any adverse events at the first administration, and she successfully obtained a negative PCR test within 1 month after diagnosis.

CAR-T therapy is an important treatment for relapsed/refractory B-cell malignancies and is being routinely administered. Therefore, the number of CAR-T cell recipients infected with SARS-CoV-2 immediately before the infusion is expected to increase. Prompt and repeated examination of PCR tests against fever or respiratory symptoms, prophylaxis with Tixagevimab−Cilgavimab, and appropriate treatment with antiviral drugs are important for the safe administration of CAR-T therapy, as in this case. However, the association between the SARS-CoV-2 anti-S-protein IgG titer and the prevention of the development of critical COVID-19 is still unclear. In addition, one of the limitations of our report is that we do not know whether SARS-CoV-2 infection, vaccination, or Tixagevimab−Cilgavimab caused the increase in the titer. More data are needed to understand the risks and the best treatment options for CAR-T cell recipients.

## Data Availability

The data that support the findings of this study are not openly available due to reasons of sensitivity and are available from the corresponding author upon reasonable request.
